# Probing stacking configurations in a few layered MoS_2_ by low frequency Raman spectroscopy

**DOI:** 10.1038/s41598-020-78238-w

**Published:** 2020-12-04

**Authors:** Rhea Thankam Sam, Takayuki Umakoshi, Prabhat Verma

**Affiliations:** 1 Department of Applied Physics, Osaka University, Suita, Osaka 565-0871 Japan; 2 PRESTO, Japan Science and Technology Agency, Kawaguchi, Saitama 332-0012 Japan

**Keywords:** Optical spectroscopy, Raman spectroscopy, Two-dimensional materials

## Abstract

Novel two-dimensional (2D) layered materials, such as MoS_2_, have recently gained a significant traction, chiefly due to their tunable electronic and optical properties. A major attribute that affects the tunability is the number of layers in the system. Another important, but often overlooked aspect is the stacking configuration between the layers, which can modify their electro-optic properties through changes in internal symmetries and interlayer interactions. This demands a thorough understanding of interlayer stacking configurations of these materials before they can be used in devices. Here, we investigate the spatial distribution of various stacking configurations and variations in interlayer interactions in few-layered MoS_2_ flakes probed through the low-frequency Raman spectroscopy, which we establish as a versatile imaging tool for this purpose. Some interesting anomalies in MoS_2_ layer stacking, which we propose to be caused by defects, wrinkles or twist between the layers, are also reported here. These types of anomalies, which can severely affect the properties of these materials can be detected through low-frequency Raman imaging. Our findings provide useful insights for understanding various structure-dependent properties of 2D materials that could be of great importance for the development of future electro-optic devices, quantum devices and energy harvesting systems.

## Introduction

Two-dimensional (2D) materials have attracted much research attention after single-layered graphene was detached from graphite by adhesive-tape technique. Graphene exhibits many interesting properties that are significantly superior than the bulk graphite^1,2^. After the success with semi-metallic graphene, layered materials with band gaps became widely sought after. Transition metal dichalcogenides (TMDCs) emerged as the new class of 2D semiconductor materials that perfectly complement graphene with a tunable bandgap^3^. TMDCs, represented by the formula MX_2_ (M = transition metal, X = chalcogen) are comprised of about 40 different layered materials such as MoS_2_, WS_2_, WSe_2_, MoSe_2_, etc. Of the TMDC family, MoS_2_ is the most studied compound and is considered as the archetypal TMDC. MoS_2_ is often used in electronic and optoelectronic devices as an active media in the form of a few layers. In 2D devices, the number of layers and their orientations in layer stacking alter the internal crystal symmetry, thereby modifying the band structure and the electronic properties of the device. For instance, bilayer graphene can be transformed into a gate-tunable superconductor or into an insulator by choosing the right twist angle between the layers^4^. It is also reported that in multilayered MoS_2_, the twist angle between the layers can change the indirect bandgap^5^. Another example is the presence of a stacking-dependent symmetry variation that leads to valley polarization, which can be utilized for valleytronics applications^6^. Natural MoS_2_ is found in two different polytypes, 2H and 3R, depending on their stacking orders. In a 2H stacked MoS_2_ with more than one layer, the intrinsic inversion symmetry hinders valley polarization. However, a 3R stacked MoS_2_ is found to exhibit a broken inversion symmetry that enhances strong inter-valley polarization, irrespective of the number of layers present. In MoS_2_ with broken inversion symmetry, such intervalley polarization can be invoked by optical excitation using circularly polarized light^7–9^. Yet another stacking dependent property present in MoS_2_ is the piezoelectricity, which arises from the broken inversion symmetry in the atomic structure as well^10^. Since 3R stacked MoS_2_ always has a broken inversion symmetry, it is expected to show piezoelectricity. In addition to stacking dependent spin–orbit coupling and symmetry variations, it has a finite bandgap that can be tuned from an indirect bandgap of 1.2 eV in the bulk sample to a direct one of 1.96 eV in the single-layer regime^3^. While the electronic and optical properties of 2D MoS_2_ are still being explored, many prototypes for nano-transistors^11^, electro-optic modulators^12^, detectors^13^, and LEDs^14^ have been developed. Since MoS_2_ has many properties depending on the layer stacking and the number of layers^15^, a precise identification of various layer stacking configurations and determination of the number of layers are essential.

Over the years, Raman spectroscopy has been proved to be one of the most versatile tools to characterize various samples, as it provides both structural and chemical fingerprints. Since it provides a non-destructive method for the determination of the number of layers, MoS_2_ has been extensively studied by Raman spectroscopy^16^. Several studies have emphasized the potential of Raman spectroscopy to study the effect of doping and defects^17,18^, as well as to measure strain in many 2D materials, including MoS_2_^19,20^. Even the subwavelength defects can be characterized by tip-enhanced Raman spectroscopy^21–27^, which enables Raman analysis with a nanoscale spatial resolution, using near-field light generated at a metallic nanotip through plasmon resonance^28–34^. Widely studied Raman modes of MoS_2_ are the high-frequency modes, E_2g_ and A_1g_, which arise due to the in-plane and the out of plane vibrations of atoms within each layer, respectively. When the number of layers increases, the frequencies of E_2g_ and A_1g_ modes are found to shift in the opposite direction^16,35^. These frequency variations are often used as indicators to identify the number of layers in MoS_2_ for up to a few layers. However, since the high-frequency modes are primarily dominated by the chemical covalent bonds, van der Waals forces between the layers have little effect on them, making them insensitive to interlayer coupling and hence to the stacking configuration of the layers.

On the other hand, the low-frequency modes that solely originate from the interlayer interactions, could be great tools to identify the stacking order and to probe interfacial qualities. In addition, they show a better response than high-frequency modes to the number layers as well, and hence should be preferred over the high-frequency modes. In the case of a few-layered MoS_2_, the reported low-frequency modes are the shear mode that originates from the in-plane oscillations of each layer; and the layer breathing mode that results from their out of plane oscillations^36^. The dependence of low-frequency modes on the number of layers was first reported in a few-layered graphene in 2012^37^. This was followed by various reports, which used the low-frequency modes to determine the number of layers^38–41^, to identify the twist angle between the layers and various stacking configurations^5,42–47^, to probe interlayer coupling^48,49^ and to determine the crystal orientation in various 2D materials and in van der Waals heterostructures^50,51^. The development of this research over the last few years indicates the ability of the low-frequency modes to characterize a wide range of 2D materials, including van der Waals heterostructures.

However, no extensive studies of the low-frequency modes to probe the stacking configurations of MoS_2_ are reported so far. Previous studies that probed stacking configuration in MoS_2_ had relied only on single point spectroscopic measurements of Raman modes^5,44,52–54^. While single point Raman measurements are sufficient to measure the properties of a homogenous sample, they do not provide an accurate picture about the distribution of any physical properties, such as the stacking configurations, within the MoS_2_ sample. A complete characterization of spatially inhomogeneous sample like an MoS_2_ flake will require imaging over the entire sample surface. For many applications, where the surface area of the active material is a crucial parameter, one needs to make sure the entire sample surface is properly characterized and studied. Determining the stacking configuration becomes even more crucial as different synthesis methods yields different degree of uniformity of stacking configuration. With Raman imaging, it is possible to extend single point Raman spectroscopy to two dimensions, which will yield much more information about the spatial variation of material properties and characteristics. In this context, Raman imaging does not only give a more comprehensive information over single point Raman measurements, but is also indispensable. As of now, no spatially resolved studies have pointed point out the subtle changes in the stacking configuration across the MoS_2_ sample. Moreover, no studies have examined the spatial variation of stacking configurations and the distribution of interfacial properties, such as twists or other defects in MoS_2_ samples. Here, we present a systematic study of few-layered MoS_2_ flakes by identifying the number of layers, stacking configurations and their distributions, and the interfacial properties in two, three, and four-layered MoS_2_ samples by analyzing the low-frequency modes through Raman imaging. Our low-frequency Raman images provides an excellent approach to assess the quality of the MoS_2_ layers and their suitability for various electronic and photonic applications.

## Results and discussions

**Figure 1:**
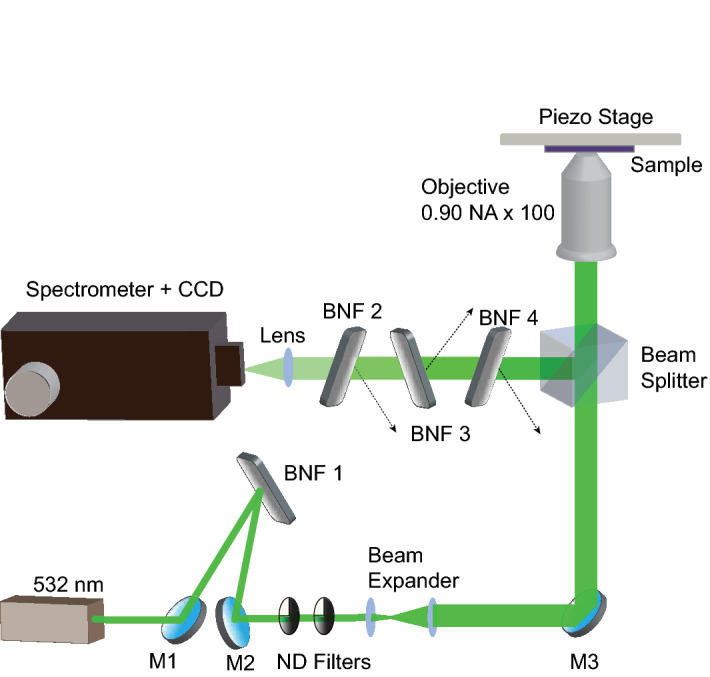
Experimental setup for low-frequency Raman measurement. M: Dielectric mirrors, BNF: Bragg notch filter, ND Filters: Neutral density filter. One BNF is used to filter the laser line, and the others are used to block Rayleigh scattered light from the sample.

The effective restoring forces associated with the low-frequency modes are quite weak as they depend only on the van der Waals interactions between the layers and are typically observed very close to the Rayleigh line. These modes are often masked by the Rayleigh line and are therefore difficult to be observed with a conventional Raman microscope. To measure the low-frequency modes, we used four different ultra-narrow band Bragg notch filters (BNFs), which have a spectral bandwidth as narrow as 4 cm^−1^. BNFs are essentially diffractive gratings that are recorded on a bulk photosensitive material by refractive index modulation through UV interference^55^. BNFs act as angular and spectral filters by reflecting an ultra-narrow bandwidth of incident light that satisfies the Bragg condition. Our experimental setup that utilizes BNFs to measure low-frequency Raman modes is illustrated in Fig. 1. Further details are described in Methods section.

**Figure 2:**
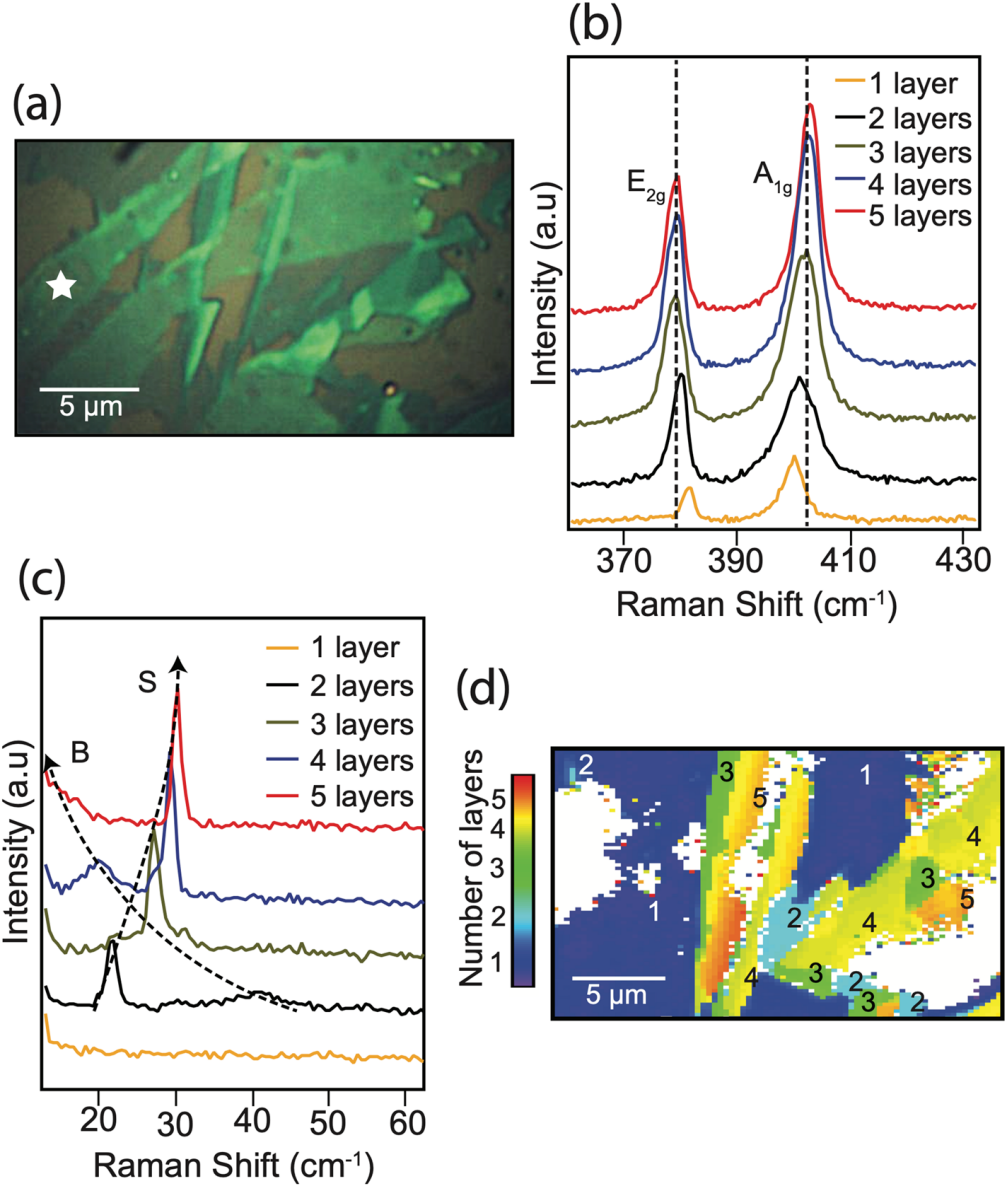
(**a**) optical image of the exfoliated MoS_2_ sample. A thin residue left by the scotch tape while exfoliation process, as confirmed from Raman measurements, is also visible in the image. The star indicates such an area, where the residues are present. (**b**) High-frequency Raman spectra collected from the MoS_2_ sample with different number of layers. (**c**) Corresponding low-frequency Raman spectra, collected from the same positions as the high-frequency spectra. The shear mode is indicated by S, while the breathing mode is indicated by B. The dashed lines show the evolution of the breathing mode and the shear mode with the increase in the number of layers. (**d**) Raman image that shows the distribution of number of layers, where the multilayered regions are constructed by the frequency shift of the shear mode and the single-layered region is constructed by the frequency differences of high-frequency modes. White regions in the image have no MoS_2_.

We used the gold-mediated tape exfoliation technique that enables exfoliation of MoS_2_ sample with the thickness of a few layers in a relatively large lateral size^56^. Figure 2(a) shows an optical image of a few layered MoS_2_ sample on SiO_2_/Si substrate prepared by Au-mediated exfoliation process. The sample preparation process is described in the Methods section. Prior to identifying the distribution of stacking configurations in the sample, we first identified the number of layers present in the sample at different locations. The high-frequency modes, E_2g_ and A_1g_, shift away from each other with increasing number of layers, which is considered as a reliable tool to determine the number of layers up-to a few layers. A Raman image showing the distribution of number of layers, constructed by the frequency difference of A_1g_ and E_2g_ modes across the area displayed in Fig. 2(a) is included in the supplementary information (Fig. S1)). E_2g_ mode redshifts while A_1g_ mode blueshifts with the increasing number of layers. With each additional layer, the increasing inter-layer van der Waals forces suppress the atomic vibrations, effectively increasing the force constants that result in the blueshift of A_1g_ mode^57^. The redshift of the E_2g_ mode is attributed to surface effects due to neighboring layers^58^ and an increase in the dielectric screening that reduces the long-range Coulombic interaction between the effective charges and thus the overall restoring forces^59^. Figure 2(b) shows Raman spectra collected from the MoS_2_ sample shown in Fig. 2(a) at locations containing different number of layers. For the number of layers up to three or four, strong signals from E_2g_ and A_1g_ with opposite shifts can be observed. However, as the number of layer increases beyond four, the changes in high-frequency modes are insignificant and are often below the instrumental resolution as they converge to the bulk value, making it difficult for an accurate estimation of the number of layers in multilayered samples. Unlike the high-frequency modes, low-frequency modes that originate from the interlayer van der Waals interactions, show higher sensitivity to the number of layers. While the frequency of the out of plane layer breathing mode decreases, the frequency of the in-plane shear mode increases with increasing number of layers. In MoS_2_, the shear mode shifts from 22 cm^−1^ in bilayer to about 32 cm^−1^ in bulk^38^. Figure 2(c) shows the low-frequency Raman spectra, measured from the same location as in Fig. 2(b) from the MoS_2_ sample shown in Fig. 2(a). Since the layers in a few-layered MoS_2_ are weakly bound as compared to the multilayer bulk MoS_2_, the overall restoring forces increase when the number of layers increases, blue-shifting the shear mode^37^. Therefore, the frequency shift of the shear mode can be used to identify the number of layers in a few-layered MoS_2_ sample. Figure 2(d) is a Raman image that shows the distribution of the number of layers in the same region as Fig. 2(a), where the multilayered regions are constructed with the frequency shift of the shear mode, and the single-layer region is constructed by the frequency differences of the two high-frequency modes A_1g_ and E_2g_. It should be noted that single-layered MoS_2_ does not have low-frequency modes because they arise from the interlayer interactions. The white regions in the image represent the SiO_2_/Si substrate, where no MoS_2_ is present. After determining the number of layers, we moved on to identify different stacking configurations present in 2-, 3-, and 4-layered regions of MoS_2_ sample by utilizing the low-frequency imaging of the layer breathing mode and the shear mode, which is discussed later.

### Layer stacking in multi-layered MoS_2_

**Figure 3:**
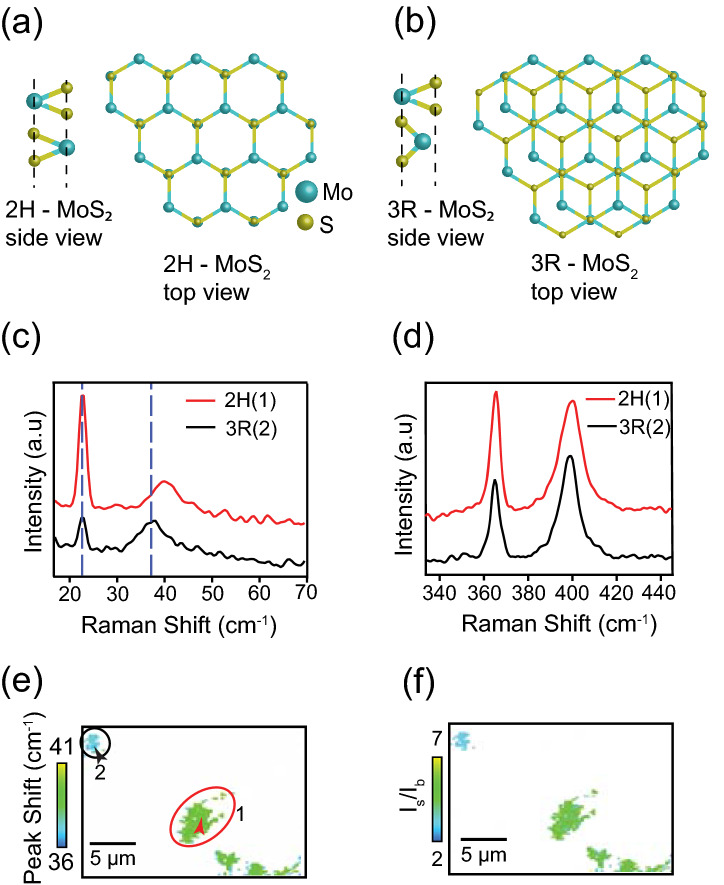
Illustrations of the side and the top views of (**a**) a 2H stacked and (**b**) a 3R stacked MoS_2_ bilayer. (b) Side and top views of a 3R-stacked two-layered MoS_2_. (**c**) Low-frequency Raman spectra representing 2H and 3R stacking configurations in a two-layered MoS_2_. (**d**) High-frequency Raman spectra, taken at the same positions as the low-frequency spectra. (**e**) Low-frequency Raman image of two-layered MoS_2_ constructed by the frequency shift of the breathing mode. The green and the turquoise colors represent 2H and 3R stacking configurations, respectively. Raman spectra in (**c**) and (**d**) are measured at the locations measured indicated by the arrow-heads in areas 1 and 2 in (**e**). (**f**) Raman image constructed by the intensity ratio I_s_/I_b_. The green and the turquoise areas represent 2H and 3R stackings, respectively. A Gaussian blurring of 1 pixel was added to the images using image processing software Fiji (https://fiji.sc/) for a better viewing^61^.

Natural MoS_2_ is found to exist in two different polytypes, 2H and 3R, depending on the sequence in which each layer is stacked. The 2H-MoS_2_ has a trigonal prismatic coordination of Mo atoms with two S-Mo-S layers connected by van der Waals forces, yielding a hexagonal lattice structure. In 2H stacking, S atoms of each layer reside directly above the Mo atoms of the lower layer masking the bottom layer (Fig. 3(a)). The 3R-MoS_2_ also has a trigonal prismatic coordination of Mo atoms, but with a rhombohedral symmetry and a different stacking order. In the case of 3R stacking, Mo and S atoms of the top layer lie above the S and Mo atoms, respectively, of the bottom layer, while the S and Mo atoms of the top layer and the Mo and S atoms of the bottom layer are situated at the center of the hexagons of the other layer (Fig. 3(b)).

### Two-layered regions

While it is already reported that every stacking configuration has its own low-frequency Raman signature and that any change in stacking configurations can affect the low-frequency Raman spectra, there are a myriad of other effects that will modify low-frequency Raman modes. The causes for such alterations include a change in the number of layers or the presence of defects. Therefore, it is made sure that the changes in the low-frequency Raman modes originated only from the changes in stacking configuration, by systematically ruling out the possibilities of the change in number of layers or the presence of defects. We have confirmed this through our parallel analysis that the number of layers is unchanged, and that there are no defects present in the sample at the measurement location. The experimental results in Fig. 3(c) show two low-frequency Raman spectra from two different two-layered regions in the sample, and Fig. 3(d) shows two high-frequency Raman spectra measured at the same location as the low-frequency modes in Fig. 3(c). While low-frequency Raman modes shows clear shifts, the simultaneously measured high-frequency Raman modes show no notable change, confirming that there is no change in the number of layers. Similarly, as reported in previous studies^18,60^, the presence of defects is expected to result in the broadening of high-frequency Raman modes as well as in activation of defect induced peaks, both of which were not observed in our measurements. Since these possibilities are ruled out, we can confirm that any changes in low-frequency Raman spectra observed in our case must have originated from a change in stacking configuration.

The spectra shown in Fig. 3(c) are identified to be originated from a 2H, and a 3R stacked bilayers, which is consistent with a previous report^58^. In the 2H stacked region, the peak around 23 cm^−1^ is identified as the in-plane shear mode, and the peak at 40 cm^−1^ corresponds to the out of plane breathing mode. In the 3R stacked region, while the shear mode stays at 23 cm^−1^, the breathing mode is found to shift to 37.4 cm^−1^, which is in good agreement with theoretical calculations^52^. The peak position of the breathing mode is red-shifted in 3R stacking, while the shear mode shows almost no change. A red-shift in the breathing mode from 2H to 3R is due to a significant decrease in the effective force constant. However for shear mode, the effective change in the force constant is very small, hence its frequency remains almost the same^58^. In order to identify two-layered regions with different stacking configurations, breathing mode can be used to construct a Raman image. The shift in the breathing mode frequency can be used as a tool to identify the differences in stacking configurations. Figure 3(e) shows a low-frequency Raman image constructed by the frequency shift of the breathing mode in the bilayered regions within the same area used in Figs. 2(a) and (d). In order to focus on the bilayered regions, only the bilayered regions in Fig. 3(e) are displayed, and the rest of the area is shown in white color. Two different colors in the image indicate the distribution of two different stacking configurations, 2H and 3R.

Further, in the two-layered region, the intensity of the shear mode is higher in the 2H stacked region as compared to the 3R stacked region, as seen in Fig. 3(c), which is also in good agreement with theoretical calculations^52^. The effect of stacking configuration on the shear mode intensity can be explained by the interlayer bond-polarizability model^62^. When exposed to the applied electric field of the incident light, the charge accumulation at the interlayer region induces dipole moments between the atoms across the layers. Raman intensities resulting from this dipole moment depend on the direction of the bond. The bond direction is determined by the atomic arrangement within each layer. When stacking configuration changes, the atomic arrangement and the effective electronic environment gets altered, thereby changing the bond direction, and varying Raman intensities. The shear mode in 2H stacking creates larger changes in polarizability due to the relative atomic positions and displacements compared to 3R stacking, hence has a higher intensity. We can, therefore, identify the 2H and 3R sequences in the bilayer region by the intensity ratio of the shear mode to the breathing mode. Since our measurements are diffraction-limited, where each measurement point is about 300 nm in diameter, the measured areas may include a few nano-sized islands of stacking sequences. In such a case, the stacking configuration can be estimated as the fraction of the two stacking sequences, 2H and 3R, for any given value of the intensity ratio. This can be obtained by interpolating the measurement between the two known values. For the 2H region, the estimated ratio of the intensity of shear mode to that of breathing mode, I_s_/I_b_, is reported to be around 4, while it was reported to be around 1 for the 3R region^54^. Any deviation from this value indicates areas with a combination of stacking sequences of 2H and 3R with certain fractions. Figure 3(f) is a Raman image of the two-layered regions as in Fig. 3(e), constructed by the intensity ratio of the shear mode to the breathing mode. Here, the green-colored region represents 2H stacking configuration (I_s_/I_b_ values close to 4), while the blue-colored region represents the 3R stacking configuration (I_s_/I_b_ values close to 1). It should be noted that the measurement of Raman intensity is associated with some measurement error, which brings in some experimental error in the value of I_s_/I_b_, resulting in an uncertainty in identifying the stacking configuration. Since our sample can have mixed stacking configurations between 2H and 3R, the stacking configurations can be identified in terms of fractions between the two configurations. For the red spectrum in Fig. 3(c), the value of the intensity ratio I_s_/I_b_, together with the possible error in measurement, is estimated 3.6 ± 3. This suggests that the measured area contains 90 ± 7% of 2H stacking and 10 ± 7% of 3R stacking. Additional low-frequency Raman spectra obtained from 2H and 3R regions are shown in Figs. S2(b) and (c), respectively, in Supplementary Information. Just as in Fig. 3(e), all the other areas except the two-layered regions are shown in white color. It should be noted that for twisted bilayers, the shear mode disappears when the twist angle is between 4—40 degrees^5,44^. In such a case, the twist angle range can be roughly estimated by the lack of shear mode and the frequency of breathing mode. Hence, the frequency shift of the breathing mode along with the intensity ratio of shear mode to that of breathing mode, I_s_/I_b_, can be used as effective tools to probe different stacking orders in a naturally stacked MoS_2_ sample.

### Three-layered regions

For more than two layers, the sample can be basically considered as two-layered MoS_2_ stacked on top of each other. In a three-layered MoS_2_, there are two parallel stacking sequences, one between layers 1 and 2, and the other between the layers 2 and 3, both of which can be the same or different at any given point. The possible stacking configurations are the 2H-2H, the 2H-3R and the 3R-3R arrangements. Depending on the position of each Mo and S atoms in every layer, 3R configuration can further have four different sub-patterns as well^42^. Three-layered regions in our sample are indicated by a lime-green color in Fig. 2(d). It should be noted that in case of a three-layered sample, DFT calculations predict two breathing modes (B1 and B2) and two shear modes (S1 and S2). However, while B2 and S2 modes are Raman inactive for 2H stacked MoS_2_, they are extremely weak for 3R stacked MoS_2_. Therefore, B1 and S1 are the only two low-frequency modes in a three-layered area that are experimentally observed for either stacking configuration^36^. Similar to two-layered regions, in three-layered areas also it is ensured that the changes in the low-frequency Raman modes are indeed originating from changes in the stacking pattern, by systematically ruling out the possibilities of any change in the number of layers and presence of defects.

**Figure 4:**
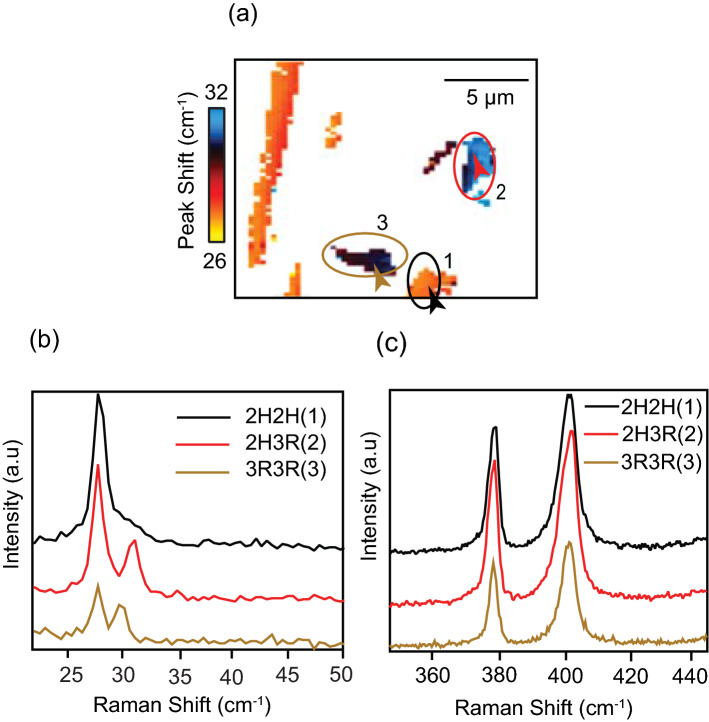
(**a**) Low-frequency Raman image of a three-layered MoS_2_ region constructed by the frequency shift of the breathing mode. Three different colors indicate the presence of three different stacking configurations. A Gaussian blurring of 1 pixel was added to the images using image processing software Fiji (https://fiji.sc/) for better viewing^61^. (**b**) Low-frequency Raman spectra measured at three locations indicated by the arrow-heads from three different areas, marked 1 to 3 in (**a**). While the region marked by number 1 indicates 2H-2H stacked regions, 2 represents 2H-3R, and 3 denotes 3R-3R regions. (**c**) High-frequency Raman spectra measured from the same positions, as the low-frequency spectra in (**b**).

In the case of the 2H-2H stacked region, the shear mode and the breathing mode have almost the same frequency, so they appear to be merged at 27 cm^−1^^54^. Similar to a two-layered region, the frequency of the breathing mode changes when the stacking configuration changes, whereas the frequency of the shear mode has no noticeable change. For locating regions with different stacking sequences, a Raman image is constructed by the frequency shift of the breathing mode, which is shown in Fig. 4(a). Only three-layered regions in Fig. 2(d) are displayed in Fig. 4(a) and the rest of the area is shown in white color. Regions with different colors in Fig. 4(a) indicate different stacking configurations. In case of three- and more layered samples, one can estimate a volume average of the fraction of stacking sequence within the diffraction-limited measurement area. Similar to the two-layered regions, the I_s_/I_b_ value is expected to be around 4 for a perfect 2H-2H configuration and 1 for a perfect 3R-3R configuration^52^. The intensity of the shear mode decreases as the stacking order changes from 2H-2H to 2H-3R and then to 3R-3R. Therefore, in order to identify distinct stacking configurations, the intensity ratio of the shear mode to the breathing mode, I_s_/I_b_, can be used. The region where the breathing mode and the shear mode are merged is identified as the 2H-2H stacked. The shear mode has maximum intensity in this region. However, since the breathing mode and the shear mode are merged in the 2H-2H region, it is difficult to estimate the exact value of the ratio, I_s_/I_b_. Therefore, an image cannot be constructed by the intensity ratio of the shear mode to the breathing mode in a three-layered sample. Figure 4(b) shows a set of low-frequency spectra taken from 3 regions, marked in Fig. 4(a), with different stacking orders. By assuming I_s_/I_b_ ratio to be around 4 for 2H and 1 for 3R stacking configurations, one can estimate an average fraction of the stacking sequence within the measurement volume from the measured value of I_s_/I_b_ at a given point. From the golden brown-colored spectrum in Fig. 4(b), the value of the intensity ratio I_s_/I_b_, together with the possible error in measurement, is estimated as 1.29 ± 0.06. This suggests that the measured area contains 90 ± 2% of 3R stacking and 10 ± 2% of 2H stacking. Similarly, from the red-colored spectrum in Fig. 4(b), the value of the intensity ratio I_s_/I_b_, together with the possible error in measurement, is estimated 2.33 ± 0.1. This suggests that the measured area contains 56 ± 4% of 3R stacking and 44 ± 4% of 2H stacking. It should be noted that I_s_/I_b_ value is the average value within the diffraction-limited focal volume. For a better understanding, additional experimental low-frequency spectra obtained from 2H-2H, 2H-3R and 3R-3R regions are shown in Figs. S3(b), (c) and (d), respectively in Supplementary Information.

The breathing mode is found to undergo a clear blue-shift as the stacking configuration changes from 2H-2H to 2H-3R. But as the configuration change from 2H-3R to 3R-3R, it is found to have red-shift, indicating a weaker interlayer interaction. The intensity and the frequency of low-frequency Raman modes serve as a unique identification tool for probing the variation in the stacking configurations in three-layered MoS_2_ as well, whereas the high-frequency Raman modes, as shown in Fig. 4(c) display little sensitivity to stacking configurations.

### Four-layered regions

**Figure 5:**
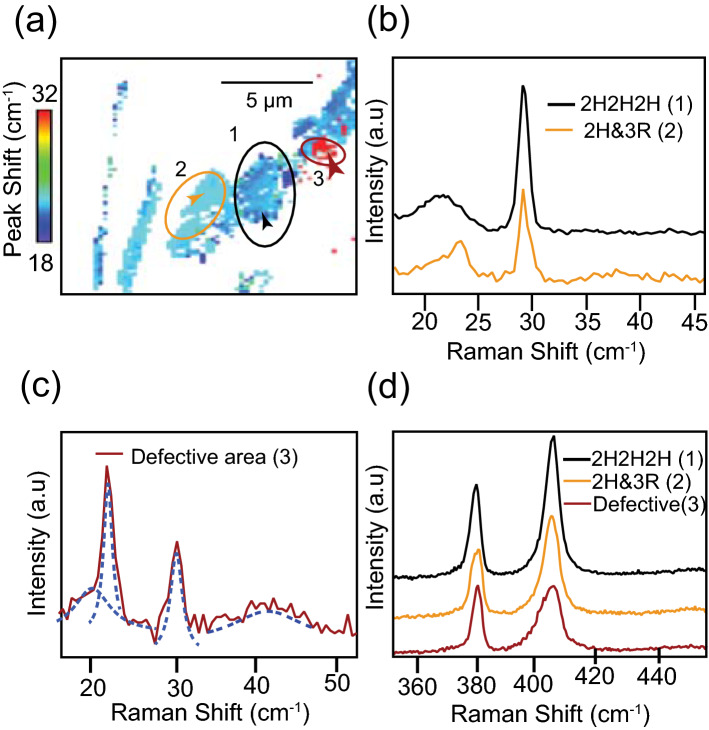
(**a**) Low-frequency Raman image of four-layered MoS_2_ regions constructed by the frequency shift of the breathing mode. Three different colors indicate the presence of three different stacking configurations. A Gaussian blurring of 1 pixel was added to the images using image processing software Fiji (https://fiji.sc/)^61^. (**b**) Low-frequency Raman spectra measured at the location of the arrowheads in the marked regions 1 and 2, marked in (**a**). (**c**) Low-frequency spectrum measured at the location of the arrowhead in the area marked 3. The dotted lines show different peaks present in the spectrum. (**d**) High-frequency spectra taken from the same positions as the low-frequency spectra shown in (**b**) and (**c**).

Similar to the case of three layers, a four-layered region of the sample can be considered as three parallel stackings of two-layered MoS_2_ on top of each other, which are between layers 1 and 2, between layers 2 and 3 and between layers 3 and 4. One can therefore find the possibility of multiple stacking sequences at one point. The stacking sequences in a four-layered MoS_2_ are much more complex than a two- or a three-layered MoS_2_. The possible stacking configurations include 2H-2H-2H, 2H-2H-3R, 2H-3R-2H, 2H-3R-3R, 3R-2H-3R, 3R-3R-3R, among many more. Since 3R can have different sub-patterns, the number of effective stacking configurations increases even more. Therefore, one can only estimate a volume average of the fraction of stacking sequence within the diffraction-limited measurement area. The four-layered region in our sample is shown in yellow color in Fig. 2(d). The most widely found stacking configuration in a four-layered MoS_2_ is 2H-2H-2H^52,63^. The shear mode for this configuration appears at 29.7 cm^−1^, and the breathing mode is found at 21 cm^−1^. Therefore, it is easy to identify the 2H-2H-2H stacking configuration in a four-layered MoS_2_^52,63^. Similar to the two- and three-layered MoS_2_, the changes in stacking configuration can be correlated to the frequency changes in the breathing mode. Figure 5(a) shows a Raman image constructed by the frequency shift of the breathing mode in the four-layered region of the sample displayed in Fig. 2(d). For convenience, only four-layered regions are displayed in Fig. 5(a) and the rest of the area is shown in white color. In the four-layered region, three different stacking configurations were identified by the frequency shift of the breathing mode, which is color-coded in Fig. 5(a).

Four-layered regions can have numerous stacking configurations, and we investigated each stacking configuration present in our sample based on the value of the I_s_/I_b_ ratio. Figure 5(b) shows spectra taken from the regions marked by 1 and 2 in Fig. 5(a), which have different stacking configurations. Similar to two- and three-layered MoS_2_, based on the I_s_/I_b_ ratio (around 4 for 2H and 1 for 3R), one can estimate an average fraction of the stacking sequence within the measurement volume at a given point. From the black-colored spectrum in Fig. 5(b), the intensity ratio I_s_/I_b_, together with the possible error in measurement, is estimated as 3.62 ± 0.2. This suggests that the measured area contains 88 ± 6% of 2H stacking and 12 ± 6% of 3R stacking. Similarly, from the yellow-colored spectrum in Fig. 5(b), the value of the intensity ratio I_s_/I_b_, together with the possible error in measurement, is estimated as 2.47 ± 0.2, suggesting the measured area contains 51 ± 6% of 2H stacking and 49 ± 6% of 3R stacking. Additional experimental low-frequency spectra obtained from 2H-2H-2H and the mixed stacking (2H&3R) areas are shown in Figs. S4(b) and (c), respectively in the supplementary information. An interesting observation here is the direction of the frequency shift of the breathing mode. In a two-layered sample, when the stacking configuration changes from 2H to 3R, the breathing mode undergoes a red-shift. In a three-layered sample, the breathing mode blue-shifts when the stacking configuration changes from a 2H-2H to a 2H-3R and then red-shifts as the stacking configuration changes from a 2H-3R to a complete a 3R-3R pattern. However, in the case of a four-layered sample, the breathing mode blue-shifts as the stacking order changes from a 2H to a 3R configuration. The reason for this behavior is presently unknown, and further investigation is required to study this behavior of the breathing mode.

Unlike the other two regions, a spectrum taken from the red-colored area in the circled region 3 shows some peculiar characteristics, which are not commonly observed in any stacking configuration. In this particular area, the appearance of multiple peaks is observed, as shown by the dotted lines in Fig. 5(c). More experimental low-frequency spectra obtained from this area is shown in Fig. S4(d) in the supplementary information. This could be due to the presence of a twist between some layers. A twist between the layers alters the atomic arrangement across the layers that causes a considerable change in the interlayer coupling. Various steps involved in the fabrication processes of the sample may cause shift and/or rotation of isolated small-sized layers, creating twisted layers in small regions. A close inspection of the spectrum reveals the presence of two breathing modes and two shear modes. The peak frequencies of these modes correspond to the peak frequencies of the low-frequency modes that appear in a four-layered and in a two-layered MoS_2_. This anomalous behavior can be explained as an overlap of two close-packed layers with another pair of close-packed layers (2 + 2) ^46^. The close-packed layers act as a single unit and create a signature of bilayers, while the overall vibration of the four layers creates the signature of four layers.

Another possible explanation for the appearance of additional modes is the presence of multiple high symmetric stacking patches at a certain twist angle between the layers^44,47^. Whenever there is a translation or rotation between layers, the stacking pattern changes accordingly. 2H and 3R stackings are interchangeable by translation. When the twist angle between the layers changes slightly, a transition of stacking patterns occurs. As the angle deviates further, the size of MoS_2_ patches with certain stacking configurations get reduced, but new patches with different stacking patterns emerge, creating a stacking configuration consisting of varying patterns. When layers are twisted at a certain angle, as shown in Fig. S5 in Supplementary Information, multiple stacking configurations can co-exist over a small area, which can even be smaller than the diffraction-limited focal spot of the incident light. Therefore, each pattern can simultaneously contribute its own characteristic low-frequency Raman peaks in a single measurement, which will result in the existence of multiple Raman peaks. The transfer process involved in sample fabrication can also create wrinkles in the layers, which will in turn modify low-frequency Raman spectra in a very similar way.

Another possibility is the presence of defects, such as atomic vacancies that can alter the interlayer interactions. Such defects are found to severely affects the high-frequency Raman modes. Figure S4(e) in Supplementary Information shows high-frequency Raman spectra taken from the red-colored region. A slight asymmetric broadening of the high-frequency mode A_1g_ suggests the co-existence of a slightly shifted mode that could be activated by the presence of atomic vacancies, originated at the edge of the Brillouin zone^18,60^. This can be identified as a disorder-induced peak and could be an indication of the presence of defects. Figure 5(d) shows high-frequency Raman spectra measured from the same position as (b) and (d).

As for the number of layers more than four, the frequency of breathing mode decreases further below 10 cm^−1^, making it undetectable due to its overlap with the Rayleigh line despite using multiple Bragg notch filters. This makes it difficult to carry out a detailed analysis of regions with more than four layers.

## Conclusions

Here, we presented an experimental approach to probe the stacking sequences in a few-layered MoS_2_ sample by low-frequency Raman imaging. These results illustrate the versatility of low-frequency modes not only for assessing the number of layers, but also to probe stacking configurations and the interlayer interactions in MoS_2_. Since spatial variations in stacking configuration is intricately linked with the synthesis technique used, point based characterization techniques need to be evolved into imaging schemes. Low frequency imaging makes it possible to map the spatial distribution of stacking configuration over the entirety of the sample. The applications outlined in this study is not limited to MoS_2_ but can be extended to other 2D materials as well. As the low-frequency modes are especially sensitive to the interlayer gap and the coupling between the layers, they have proved to be valuable in detection of uneven interfaces caused by strain, layer twists or defects. Recent breakthroughs in the domains of twistronics, valleytronics and nanoelectronics have been assisted by a detailed understanding of 2D layered materials regarding their uniformity and defect levels. Through this study, we have demonstrated low-frequency Raman imaging to be one of the best tools available to easily and robustly characterize the sample by detecting its uneven inter-facial characteristics.

## Materials and methods

### Sample preparation

A 100-nm-thick gold film was grown on a thin multilayered MoS_2_ flake that was exfoliated using scotch tape from a bulk crystal. A thermal release tape was used to peel off the Au layer along with MoS_2_ layers beneath it, which was then transferred to the SiO_2_/Si substrate. The thermal release tape was released by heating the substrate along with the tape on a hotplate. Gold has a strong affinity for chalcogen atoms and is known to form a semi-covalent bond with sulfur atoms. The interaction between Au and the topmost MoS_2_ layer is stronger than the van der Waals interaction between the same MoS_2_ layer and the layers under it, which ideally enables a selective removal of the topmost layer. However, since the interaction between any two neighboring MoS_2_ layers is weaker than the interaction between Au and the topmost MoS_2_ layer, it is possible that a few layers of MoS_2_ are exfoliated, together with the topmost layer. The Au film is subsequently etched off using KOH/I_2_ solution. The color contrast in Fig. 2(a) represents the variation in the number of layers across the sample area.

### Raman measurements

A 532 nm laser was focused onto the MoS_2_ sample by a 100 X objective lens with an NA of 0.90. The laser power was kept around 1 mW on the sample. The acquisition time was kept at 900 ms to ensure a good signal-to-noise ratio. Measurements are done with a spectral resolution of 0.5 cm^-1^ and a spatial resolution of about 300 nm. The scattered light was collected and dispersed by a spectrometer from Princeton Instruments (Acton SP2300, Acton, USA), equipped with a grating of 1800 grooves/mm and finally detected by an EM-CCD camera from Princeton Instruments (Pixis 100, Acton, USA). Bragg notch filters from Optigrate were used to filter the laser and to reject the Rayleigh scattered light. BNF-1 in Fig. 1 is used as a filter to reject any possible unwanted wavelengths in the laser beam, while BNF-2, 3 and 4 reject the Rayleigh scattered light. The tilt angles of the BNFs are adjusted to meet the Bragg condition to provide maximum attenuation of the Rayleigh scattered light. The dashed arrows in Fig. 1 indicates the direction of reflected light. Unlike BNF-2 and -4, the tilt angle of BNF-3 is chosen to be different to change the direction of the reflected light for minimizing the multiple reflections between the filters.

## Supplementary information

Supplementary Information.

## Abbreviations

2D: Two-dimensional
TMDC: Transition metal dichalcogenide
S: Shear mode
B: Breathing mode
