# Supplementary material for: Probing stacking configurations in a few layered MoS2 by low frequency Raman spectroscopy
Source: Sci Rep. 2020 Dec 4;10:21227. doi: 10.1038/s41598-020-78238-w (PMC7718217; doi:10.1038/s41598-020-78238-w)
Supplement: Supplementary file 1 — Supplementary Information. [file 41598_2020_78238_MOESM1_ESM.pdf]

Supplementary Information

Probing stacking configurations in a few layered MoS_2 by low frequency Raman spectroscopy

Rhea Thankam Sam[1], Takayuki Umakoshi[1,2] and Prabhat Verma[*,1]

[1]Department of Applied Physics, Osaka University, Suita, Osaka 565-0871, Japan
[2]PRESTO, Japan Science and Technology Agency, Kawaguchi, Saitama 332-0012, Japan
[*]Email: verma@ap.eng.osaka-u.ac.jp

Identification of number of layers using high-frequency Raman modes.

For a better confirmation of the number of layers, we utilized the high-frequency modes A_{1g} and E_{2g}, in addition to using the frequency shift of the low-frequency shear mode. When the number of layers increases, the A_{1g} mode blueshifts, whereas the E_{2g} mode undergoes a redshift. Since they shift in opposite directions, the difference in their frequencies can be used as a tool to map the number of layers. Figure S1 shows a Raman image constructed by the frequency difference of A_{1g} and E_{2g} modes, indicating the distribution of the number of layers.

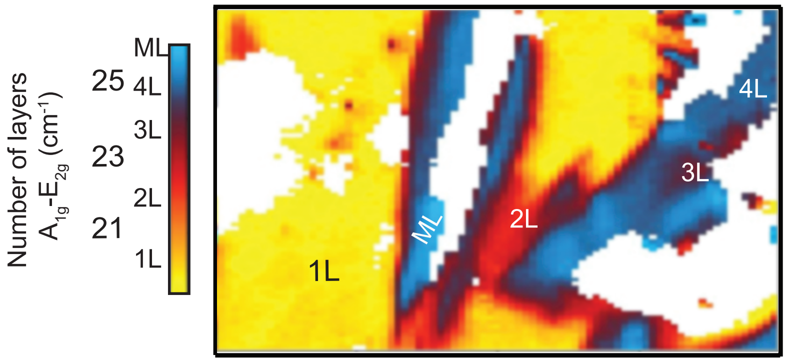

Figure S1. Raman image constructed by the frequency differences of A_{1g} and E_{2g} modes that shows the distribution of number of layers across the few-layered MoS_2 sample. The number of layers varies from a single-layer (1L) to more than four layers, which is marked as multilayers (ML). White regions in the image have no MoS_2.

Additional Raman spectra measured from two-layered regions in the MoS_2 sample

Same as in Fig. 3(e) in the paper, Fig. S2(a) shows a Raman image constructed by the frequency shift of the breathing mode, which shows the distribution of the stacking configurations across

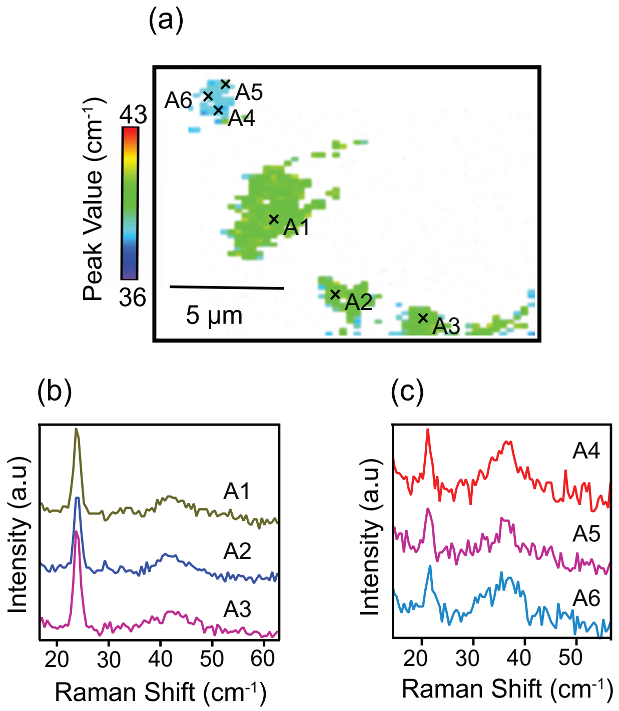

Figure S2. (a) Raman image constructed by the frequency shift of the breathing mode that shows the distribution of stacking configurations across the two-layered regions in the MoS_2 sample, same as in Fig. 3(e). Low-frequency Raman spectra measured (b) within the 2H stacked area at locations A1, A2 and A3; and (c) within the 3R stacked area at locations A4, A5 and A6.

the two-layered regions in our sample. Here we show some additional points within the two-layered MoS_2. Figure S2(b) shows three different Raman spectra collected from points A1, A2, and A3 that lie within the green-colored areas in Fig. S2(a). These spectra are taken from the 2H stacked area of the sample. As observed, low-frequency modes show similar characteristics throughout the 2H stacked area. Similarly, Fig. S2(c) shows three different Raman spectra collected from points A4, A5, and A6 within the blue-colored areas in Fig. S2(a). These spectra are taken from the 3R stacked area of the sample. They also show similar behavior throughout the 3R stacked area as reported in the paper.

Additional Raman spectra measured from three-layered regions in the MoS_2 sample

In order to provide additional information, we investigated different three-layered regions by considering the behavior of Raman spectra at several locations marked by B1 through B9 in Fig. S3(a), which is the same Raman image displayed in Fig. 4(a), constructed by the frequency shift of the breathing mode showing the distribution of the stacking configurations across the three-layered region in our sample. The low-frequency Raman spectra measured at locations B1, B2 and B3 that lie within the orange-colored region (2H-2H stacking) are displayed in Fig. S3(b). Similarly, the low-frequency Raman spectra measured at locations B4, B5 and B6 that lie within the blue-colored region (a combination of 2H&3R stacking) are displayed in Fig. S3(c), and the low-frequency Raman spectra measured at locations B7, B8 and B9 that lie within the brownish-black colored region (3R-3R stacking) are displayed in Fig. S3(d). The frequencies and intensities of the low-frequency Raman modes within a particular stacking configuration are very similar.

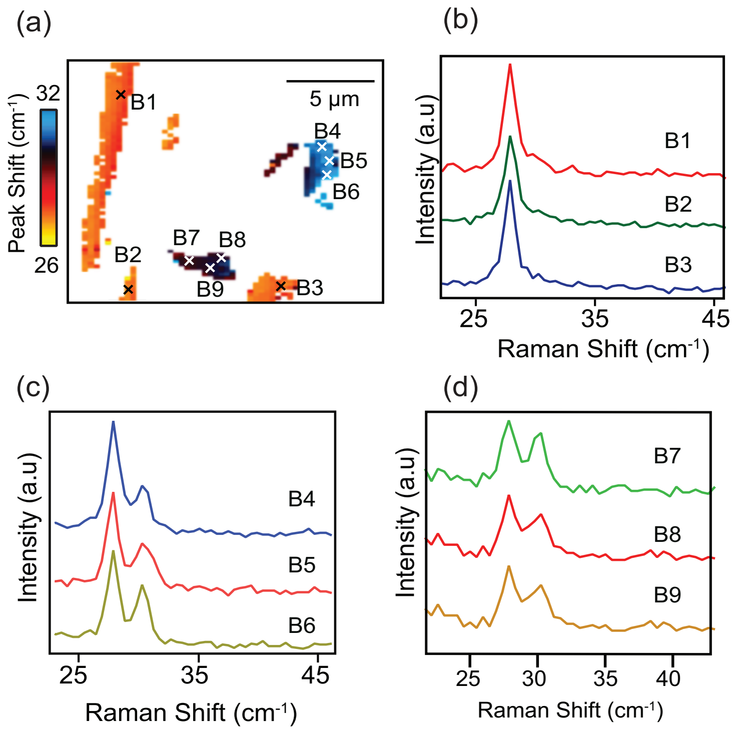

Figure S3. (a) Raman image constructed by the frequency shift of the breathing mode that shows the distribution of stacking configurations across the three-layered region in the MoS_2 sample, same as in Fig. 4(a). Low-frequency Raman spectra measured (b) within the 2H-2H stacked areas at locations B1, B2 and B3; (c) within the 2H-3R stacked areas at locations B4, B5 and B6; and (d) within the 3R-3R stacked areas at locations B7, B8 and B9.

Additional Raman spectra measured from four-layered regions in the MoS_2 sample

Further, we investigated four-layered regions in the MoS_2 sample by analyzing the behavior of low-frequency Raman spectra at several locations marked by C1through C9 in Fig. S4(a), which is the same Raman image displayed in Fig. 5(a), constructed by the frequency shift of the breathing mode showing the distribution of the stacking configurations across four-layered region in our sample. The low-frequency Raman spectra within the dark-blue colored region, which appears to have mostly 2H-2H-2H stacking (C1, C2, and C3), the turquoise-colored area with a mixed stacking area of 2H&3R (C3, C4, and C5), and the defective area (C7, C8, and C9) are displayed in Figs. S4(b), S4(c) and S4(d), respectively. These spectra measured from the regions with 2H-2H-2H stacking, mixed stacking of 2H&3R and the defective area have their own distinct features as reported in the paper. However, a detailed analysis of the high-frequency Raman modes measured within the red-colored defective region in Fig. S4(a) shows some interesting features. Figure S4(e) shows high-frequency Raman spectra measured within the defective area (the red-colored region in Fig. S4(a)) at locations C7, C8, and C9 (taken from the same positions as the low-frequency spectra shown in Fig. S4(d)), along with another spectrum, C1 measured outside the defective area for comparison. The Lorentzian fits of A_{1g} mode in Fig. S4(e) show the co-existence of a slightly shifted mode that could be activated by the presence of atomic vacancies, originated at the edge of the Brillouin zone. This could be an indication of the presence of defects and it can be identified as a disorder-induced peak.

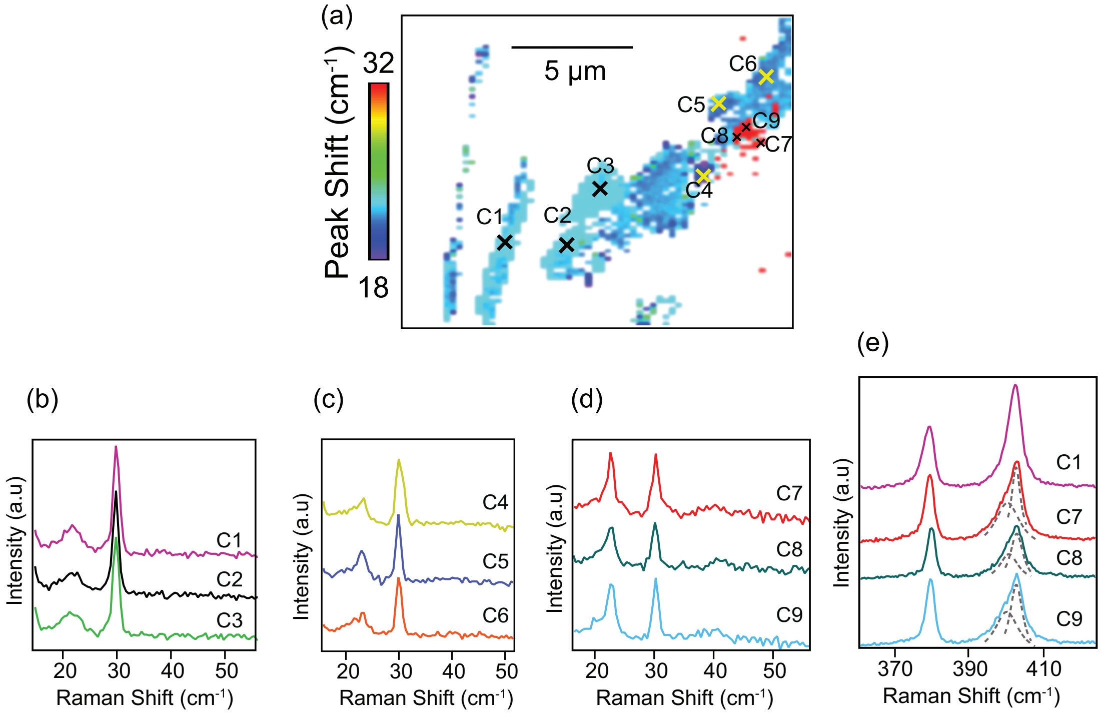

Figure S4. (a) Raman image constructed by the frequency shift of the breathing mode that shows the distribution of stacking configuration across the four-layered region in the MoS_2 sample (same as in Fig. 5(a)). Low-frequency Raman spectra measured (b) within the 2H-2H-2H stacked area at locations C1, C2, and C3; (c) within the mixed stacked area (2H-3R) at locations C4, C5, and C6; and (d) within the defective area (red-colored region) at locations C7, C8, and C9 showing multiple low-frequency peaks. (e) High-frequency Raman spectra measured within the defective area (the red-colored region in Fig. 5(a)) at locations C7, C8, and C9, along with the spectrum C1, measured outside the defective area for comparison. A careful look at the A_{1g} mode at C7, C8, and C9 reveals another peak towards the lower frequency side of the peak. The grey dashed-lines are the Lorentzian fits used to reveal the second peak.

Twisted MoS_2 layers

A representation of a twisted bilayer MoS_2 is shown in Fig. S5, where the top layer is twisted at an angle α with respect to the bottom layer. When layers are twisted at a certain angle, as shown in Fig. S5, multiple stacking configurations, shown by the black dotted circles, can co-exist over a small area, which can even be smaller than the diffraction-limited focal spot of the incident light, shown by the light-blue circle in the figure. Therefore, each pattern can simultaneously contribute its own characteristic low-frequency Raman peaks in a single measurement, which will result in the existence of multiple Raman peaks.

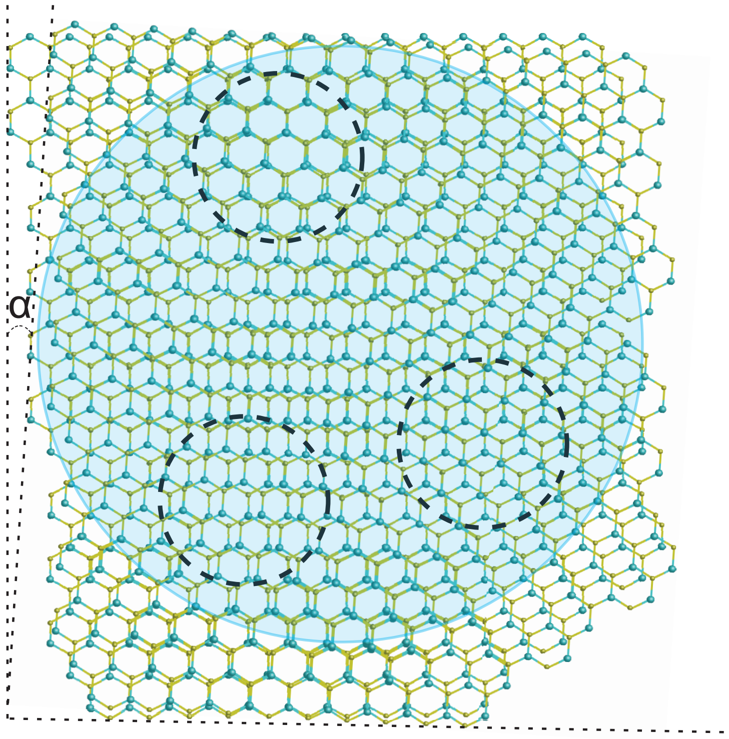

Figure S5. A twisted MoS_2 sample, where the top layer is twisted at an angle α with respect to the bottom layer. Patches with different stacking configurations are marked by the black dotted circle. Large light-blue circle represents the focus spot of the incident light.
